# Can Interrogation of Tumour Characteristics Lead us to Safely Omit Adjuvant Radiotherapy in Patients with Early Breast Cancer?

**DOI:** 10.1016/j.clon.2017.12.022

**Published:** 2018-03

**Authors:** I.S. Bhattacharya, A.M. Kirby, J.M. Bliss, C.E. Coles

**Affiliations:** ∗ Institute of Cancer Research, Clinical Trials and Statistics Unit, London, UK; † Department of Radiotherapy, The Royal Marsden NHS Foundation Trust, Sutton, Surrey, UK; ‡ University of Cambridge, Oncology Centre, Cambridge University Hospitals NHS Foundation Trust, Cambridge, UK

**Keywords:** Biomarker, breast, cancer, clinical, de-escalation, radiotherapy, trials

## Abstract

Adjuvant radiotherapy after breast-conserving surgery has been an important component of the standard of care for early breast cancer. Improvements in breast cancer care have resulted in a substantial reduction in local relapse rates over recent decades. Although the proportional benefits of adjuvant radiotherapy are similar for different prognostic risk groups of patients, the absolute benefits depend on the risk of relapse and therefore vary considerably between prognostic groups. Radiotherapy is not without risk and for some patients at very low risk of relapse the risks of radiotherapy may outweigh the benefit, leading to potential overtreatment.

Randomised controlled trial (RCT) evidence shows that omission of radiotherapy in low risk early breast cancer does not reduce overall survival or increase breast cancer mortality and local recurrences are salvageable. Despite this there has not been a change in practice regarding omission of radiotherapy. The reasons for this may include challenges in patient selection. Recent advances in immunohistochemistry and genomic profiling may improve risk stratification and the development of biomarkers to directed therapies. Several RCTs have quantified the benefit of radiotherapy in reducing local relapse. Where a treatment benefit is known but is considered to be so small not to be clinically relevant then alternatives to RCTs may be considered to answer the question of need. This is because we can assess risk against a fixed ‘absolute’ boundary rather than needing a randomised comparator. The prospective cohort study is an alternative to the RCT design to answer the question of need for radiotherapy. The feasibility of recruitment into biomarker-directed de-escalation studies will become apparent as more studies open. The challenge is to determine if we are able to accurately risk stratify patients and avoid unnecessary toxicity, thereby tailoring the need for adjuvant breast radiotherapy on an individual patient basis.

## Statement of Search Strategies Used and Sources of Information

MEDLINE, Pubmed, EMBASE and the Cochrane library were searched in 2017 for relevant literature on biomarker-directed avoidance of radiotherapy studies. The National Institutes of Health and ClinicalTrials.gov databases were searched in 2017 for relevant ongoing and unpublished clinical trials.

## Introduction

Adjuvant radiotherapy after breast-conserving surgery (BCS) has been shown to reduce the risk of a recurrence by one-half and breast cancer mortality by one-sixth in patients with early breast cancer [1]. The absolute benefit of radiotherapy is dependent on the individual's risk of relapse and can vary substantially for different prognostic risk groups of patients [1]. Radiotherapy is not without risk and this risk is dependent on factors other than breast cancer prognosis. The risks of radiotherapy may outweigh the benefit for some women at very low risk of breast cancer relapse. This overview examines the challenges and novel approaches to de-escalating breast radiotherapy through clinical research studies.

## What are the Factors Contributing to the Risk of Local Relapse?

Meta-analysis data of patients in trials of adjuvant radiotherapy after BCS suggest that local recurrence risk depends strongly on nodal status and in node-negative patients, young age, poor tumour differentiation and large tumour size indicate a high local recurrence risk [1], [2]. A recently published multi-institutional cohort of 2233 consecutive breast cancer patients who underwent BCS and postoperative radiotherapy between 1998 and 2007 observed 69 local recurrences with a median follow-up of 106 months [3]. Non-luminal A subtypes (hazard ratio for luminal B 2.64, *P* = 0.001, for HER2-positive 5.42, *P* < 0.0005 and triple-negative breast cancer 4.33, *P* < 0.0005), age ≤ 50 years (hazard ratio 0.56 for patients older than 50 years; *P* = 0.01) and increasing nodal involvement (hazard ratio 1.06 per involved node, *P* = 0.004) were independent risk factors for increased local recurrence on multivariate analysis. Of note, high histological grade (hazard ratio 5.37, *P* < 0.001), T3 disease (hazard ratio 10.39, *P* < 0.001) and positive margins (hazard ratio 2.43, *P* = 0.005) were significantly associated with increased risk of local recurrence on univariate but not on multivariate analysis. Identifying risk factors for local recurrence may help to determine when adjuvant radiotherapy is required.

## What are the Benefits of Adjuvant Breast Radiotherapy?

Historical data from the Early Breast Cancer Trialists' Collaborative Group (EBCTCG) analysis of >10 000 patients randomised into trials of BCS with and without radiotherapy have shown radiotherapy to the conserved breast halves the rate at which a disease recurs and reduces the breast cancer death rate by about a sixth [1], [4]. There have been a number of improvements in breast cancer care and a reduction in local relapse rates than those reported in trials on which the EBCTCG meta-analysis was based [5]. Earlier cancer detection, improvements in the quality and standardisation of surgery, developments in systemic therapies and radiation techniques may have contributed to the reduced rates of local relapse [5]. Although the relative benefit from breast radiotherapy remains the same, the absolute benefit is much smaller by virtue of the decreased local relapse rate. As breast cancer survival increases, the late permanent effects of radiotherapy become more apparent and greater patient advocate voice and survivorship awareness have highlighted the problems patients face regarding long-term adverse effects.

## What are the Risks of Adjuvant Breast Radiotherapy?

Despite advances in radiation techniques, rare life-threatening side-effects may occur. A large case–control study in 2168 patients showed an increased rate of major coronary events by 7.4%/Gy mean heart dose with breast radiotherapy, with no apparent ‘safe’ threshold dose to the heart [6]. The absolute risk of radiation-induced cardiac toxicity increases considerably in patients with pre-existing cardiac risk factors [6]. A meta-analysis that included >700 000 women showed that breast radiotherapy was significantly associated with an additional second cancer risk, the highest being second lung cancer risk (relative risk 1.66; 95% confidence interval 1.36–2.01) and the second in incidence was second oesophageal cancer risk (relative risk 2.17; 95% confidence interval 1.11–4.25) that increased over time at least 15 years after treatment [7]. A meta-analysis of trials of women randomly assigned to radiotherapy versus no radiotherapy yielded a lung cancer incidence ≥10 years after radiotherapy rate ratio of 2.10 (95% confidence interval 1.48–2.98; *P* < 0.001) and for cardiac mortality, the rate ratio was 1.30 (95% confidence interval 1.15–1.46; *P* < 0.001). Smoking was found to determine the net effect of radiotherapy on mortality [8].

More commonly, radiotherapy can lead to normal tissue effects affecting the treated breast. For example, the 10 year analysis of the UK START trials reported moderate/severe chronic adverse effects, including breast shrinkage, pain, tenderness and hardness [9], leading to impaired quality of life and psychological distress [10].

Given the potential risk of toxicity associated with adjuvant breast radiotherapy there is an increasing view among clinicians that in patients at very low risk of local relapse the side-effects of radiotherapy may outweigh the benefits.

## What is the Evidence to Date?

Several studies have randomly assigned women with early breast cancer to receive hormonal therapy with or without radiotherapy and have shown small but significantly improved local control rates in patients receiving radiotherapy [11], [12], [13], [14], [15].

The Cancer and Leukaemia Group (CALGB) and PRIME II trials recruited women over 70 and 65 years, respectively. Fyles *et al.* [11] recruited women >50 years, but almost three-quarters of women were aged >60 years. The BASO II trial recruited women <70 years. There is no agreed age cut-off as to what constitutes an older patient.

The CALGB 9943 trial randomly assigned 636 women ≥70 years with stage I oestrogen receptor-positive disease and tumour size ≤2 cm to receive BCS and tamoxifen with or without radiotherapy and showed that radiotherapy did not improve 5 year overall survival or disease-free survival or decrease the rate of mastectomy for recurrence. In patients receiving radiotherapy there was a small but statistically significant improvement in local relapse. Local relapse was 1% (95% confidence interval 0–2%) in patients receiving radiotherapy versus 4% (95% confidence interval 2–7%, *P* < 0.001) in patients not receiving radiotherapy [13]. The CALGB 10 year local recurrence rates were 2% (95% confidence interval 1–4%) and 9% (95% confidence interval 6–13%) for those who did and did not receive radiotherapy, respectively. Further analysis at 10 years showed no difference in overall survival or breast cancer-specific deaths in those who received radiotherapy (67%; 95% confidence interval 62–72%) and those who did not (66%; 95% confidence interval 61–71%) [16].

The National Comprehensive Cancer Network amended its guidance stating that adjuvant radiotherapy may be omitted in patients with a low risk of local relapse after publication of the CALGB 5 year data [17]. Despite reporting no excess of distant relapse or increase in breast cancer-related deaths and showing that local relapses may be salvaged with surgery ± radiotherapy, ‘omission of radiotherapy’ was not adopted into clinical practice [18]. A subsequent analysis of Medicare patients meeting the eligibility criteria of the CALGB study showed that the use of radiotherapy only reduced from 79% to 75% of patients in the general population who met the trial eligibility [19]. There are a number of possible reasons why this trial did not bring about a substantial change in practice. Some clinicians may have felt that a median follow-up of 5 years was insufficient to advocate a change in practice [19]. Others may have found patient selection for no radiotherapy challenging given the lack of information regarding histology, grade, margin status or presence of lymphovascular invasion recorded within the trial. There may have been some concerns that monitoring of endocrine therapy compliance could be less rigorous outside the setting of a clinical trial, resulting in higher relapse rates [18]. Some opponents have also argued that in a slow growing breast cancer there may be a long interval between the onset of a recurrence and recurrence-related mortality and provided sufficient time is allowed mortality will be increased [20]. Conservatism in the clinical community is also a factor. Financial benefits and higher reimbursement may also contribute to clinicians preferring to opt for treatment [19]. Patient preference may play a role, with some women still opting to receive radiotherapy to minimise the risk of local relapse, despite the lack of survival benefit.

More recently, the PRIME II study randomly assigned 1326 women aged ≥65 years with oestrogen receptor-positive disease, tumour size ≤3 cm pN0 tumours to receive BCS and endocrine therapy with or without radiotherapy [14]. At a median follow-up of 5 years, similar local control rates to CALGB were shown in the radiotherapy (1.3%; 95% confidence interval 0.2–2.3) versus no radiotherapy groups (4.1%; 95% confidence interval 2.4–5.7; *P* = 0.0002) and there was no reported excess of distant relapse, second cancers or deaths. The study showed that local relapses may be salvaged with surgery ± radiotherapy without increasing the risk of breast cancer death in both groups (5 year overall survival 93.9%, confidence interval 91.8–96%; *P* = 0.34) [14]. In an unplanned subgroup analysis, oestrogen receptor-rich patients receiving endocrine treatment and radiotherapy had only a 2.4% absolute gain in local relapse over patients receiving endocrine treatment alone. The local relapse with radiotherapy was 0.8% (95% confidence interval 0.3–1.9) versus 3.2% (95% confidence interval 2.1–5.2) with no radiotherapy. Additionally, in a study conducted by Fyles *et al.* [11], a planned subgroup analysis of 611 women with T1, oestrogen receptor-positive tumours indicated a benefit from radiotherapy (5 year rates of local relapse, 0.4% with tamoxifen plus radiotherapy and 5.9% with tamoxifen alone; *P* < 0.001). Liu *et al.* [21] carried out intrinsic subtyping on tissue banked from the Fyles *et al.* study and found a low rate of local recurrence in luminal A patients with or without radiotherapy.

This suggests there may be a group of women with a very low risk of local recurrence where adjuvant radiotherapy could be de-escalated. However, improved techniques above basic clinicopathological factors are required to select this group of patients.

## How can we Identify an Individual Patient's Risk of Relapse?

The CALGB and PRIME II studies show that basic clinicopathological parameters including T1/N0/oestrogen receptor-positive, grade 1/2 and older patient age may broadly categorise a group of patients with an anticipated low 5 year local relapse rate without radiotherapy. However, improved selection of individual patients at very low risk of relapse is required before widespread change in clinical practice can be advocated. Modern molecular diagnostics may improve the estimation of relapse risk for individual patients and gene profiling and immunohistochemistry (IHC) techniques may be used as biomarkers to direct treatment.

The Oncotype DX 21 gene recurrence score was developed to categorise early breast cancer patients into risk categories for distant recurrence and has been validated in the tamoxifen and anastrazole monotherapy groups of the Arimidex, Tamoxifen Alone or Combined (TransATAC) study [22]. The recurrence score was found to improve risk stratification in postmenopausal patients in the TransATAC study [22]. When incorporating classical clinicopathological parameters (the clinical treatment score) the prognostic precision of the Oncotype DX recurrence score was improved [23], [24]. A significant association between recurrence score and the risk for locoregional recurrence was found in patients with node-negative, oestrogen receptor-positive breast cancer from two National Surgical Adjuvant Breast and Bowel Project (NSABP) trials (NSABP B-14 and B-20), similar to the association between recurrence score and risk of distant recurrence [25].

The Prosigna assay centred on the PAM50 gene signature was developed to identify intrinsic breast cancer subtypes (luminal A/B, HER2 enriched, basal like) and a risk of recurrence (ROR) score that correlates with the probability of distant recurrences [26], [27]. The Prosigna ROR was found to add significant prognostic information over standard clinicopathological parameters in both the Austrian Breast and Colorectal Study Group (ABCSG-8) trial [28] and TransATAC trials [29]. A combined analysis of these two trials showed that ROR predicted late distant recurrence beyond clinicopathological parameters [30]. Prosigna ROR was also used to predict local recurrence-free survival (LRFS) in patients randomised within ABCSG-8 comparing tamoxifen versus tamoxifen and radiotherapy after BCS. Prosigna ROR and intrinsic subtype were independent predictors of LRFS [31]. The genomic expression test EndoPredict was also tested in the ABCSG-8 trial and was an effective prognostic tool for predicting LRFS, but among postmenopausal, low-risk patients, EndoPredict did not seem to be useful for tailoring local therapy [32].

As well as genomic profiling, advances have been made in the development of IHC techniques. IHC4+Clinical (IHC4+C) combines the expression of oestrogen receptor, progesterone receptor, HER2 and Ki-67 with clinicopathological parameters (tumour size, grade, nodal status, age and endocrine treatment) to identify breast cancer patients at very low, low, intermediate or high risk of distant disease recurrence [23]. IHC4+C was developed using data from the ATAC study [33] and has subsequently been validated on another cohort [23]. In a comparison of Prosigna ROR, Oncotype DX and IHC4+C for predicting risk of distant recurrence after endocrine therapy in the TransATAC study, IHC4+C provided comparable prognostic information when compared with the Prosigna ROR and more accurate prognostic information when compared with Oncotype DX [29]. In addition, one study found a significant association between IHC4+C and risk of locoregional recurrence in postmenopausal women with oestrogen receptor-positive early breast cancer in patients who did not receive adjuvant radiotherapy [34].

Further investigation and prospective validation of IHC and genomic profiling techniques in determining the risk of locoregional relapse is required. The financial implications of using these biomarkers need consideration. IHC4+C may be a preferable biomarker in certain healthcare systems as it is cost-effective when compared with Oncotype DX and Prosigna, as it can be calculated from parameters used in routine clinical practice without excessive increases in cost.

## Current De-escalation of Radiotherapy Studies

Several randomised controlled trials (RCTs) involving 8000 women and >10 years of follow-up have quantified the effect of radiotherapy in reducing local relapse after BCS for early breast cancer [2]. The challenge is now to identify the very low risk population where even if radiotherapy is omitted, the rate of local relapse will be very low and the side-effects of radiotherapy would be predicted to outweigh the benefits. In order to recruit to an RCT both patients and clinicians need to be uncertain of the benefit of radiotherapy. Conducting RCTs testing treatment versus no treatment may be a challenge to implement as patients may have strong preferences regarding treatments. The RCT design does, however, enable the investigation of potential radiosensitivity signatures. The PRIME study randomised women to receive radiotherapy or not after BCS [37]. Patient accrual was challenging, particularly as patients did not want to be randomised and the trial design was amended allowing non-randomised patients who requested no radiotherapy to be followed up within a cohort design, which improved recruitment.

The prospective cohort design concentrates specifically on the need for radiotherapy in a population considered to be at such a low risk of recurrence that the potential absolute gain from radiotherapy is considered so small that the risks outweigh that benefit. The purpose of this trial design is to compare an observed event rate within a cohort with a fixed incidence considered to be at the upper limit of acceptability, to identify the need for the intervention, i.e. radiotherapy. This study design avoids randomisation and may facilitate rapid accrual. In an RCT, the event rates of the two groups of patients are compared, whereas in a cohort study the event rate in the cohort is compared with a prespecified cut-off.

A number of prospective biomarker-directed studies exploring the de-escalation of radiotherapy are currently recruiting in various countries. The PRIMETIME [38], LUMINA [39], IDEA [40] and PRECISION [41] studies have all used the biomarker-directed prospective cohort designs, whereas the EXPERT trial [42] has adopted a biomarker-directed RCT design (see Table 1). These studies aim to generate evidence supporting de-escalation of adjuvant radiotherapy in a population of patients with such a low risk of local relapse that the risks of radiotherapy outweigh the benefit. The primary end point for each of these studies is local recurrence at 5 years. All participants will have had BCS and receive standard endocrine therapy.

**Table 1:** Summary of biomarker-directed ‘avoidance of radiotherapy’ studies

Study name (date opened)	Country of origin	Study design	Eligibility criteria (age)	Margin requirement after breast-conserving surgery	Eligibility criteria (T1, grade 1–2, ER/PR-positive HER2-negative, node-negative)	Eligibility criteria (additional)	Anticipated ipsilateral recurrence rate	Expected recruitment (patient number)
PRIMETIME (May 2017) [38]	UK	Prospective cohort	≥60 years∗	≥1 mm microscopic, circumferential margins of normal tissue from invasive cancer and DCIS	✓	Ki-67 to determine IHC4+C	≤4% at 5 years	1500
LUMINA (July 2013) [39]	Canada	Prospective cohort	>55 years	≥1 mm microscopically clear resection margins for invasive disease and DCIS or no residual disease on re-excision	✓	IHC including ER/PR/HER2, Ki-67 to determine luminal A subtype	<5% at 5 years <10% at 10 years	500
IDEA (March 2015) [40]	USA	Prospective cohort/single group assignment	50–69 years	Margins of excision ≥2 mm	Also included grade 3	Oncotype-DX RS ≤ 18	<6% at 5 years	200
PRECISION (May 2016) [41]	USA	Phase II prospective cohort	50–75 years	Negative margins (‘no ink on tumour’) or re-excision showing no residual disease in the re-excision specimen	✓	PAM-50 (luminal A subtype, low-risk ROR)	<5% at 5 years	690
EXPERT (August 2017) [42]	Australia and New Zealand	Randomised controlled trial	≥50 years	Microscopically negative margins for invasive carcinoma and any associated DCIS (no cancer cells adjacent to any inked edge/surface of specimen) or re-excision showing no residual disease	✓	PAM-50 (luminal A subtype, ROR ≤60)	≤4% at 5 years	1170

DCIS, ductal carcinoma *in situ*; ER, oestrogen receptor; PR, progesterone receptor; RS, recurrence score; IHC4+C, IHC4+Clinical; ROR, risk of recurrence.

∗ Younger patients are eligible if they are postmenopausal and have comorbidities that imply a high risk of radiotherapy toxicity.

The PRIMETIME study ‘Post-operative avoidance of radiotherapy: biomarker selection of women categorised to be in a very low risk group by IHC4+C’ being led in the UK is using IHC4+C (incorporating Ki-67) to direct treatment [38]. The LUMINA study ‘A prospective cohort study evaluating risk of local recurrence following BCS and endocrine therapy in low risk luminal A breast cancer’ is also using IHC, including oestrogen receptor, progesterone receptor, HER2 and Ki67 status to determine luminal A subtype and direct treatment [39]. Of note, the LUMINA study will not incorporate the clinical factors used in IHC4+C.

The University of Michigan Cancer Centre is leading the IDEA ‘Individualized decisions for endocrine therapy alone’ study, which is a single group assignment study using the biomarker Oncotype DX [40]. The PRECISION ‘Profiling early breast cancer for radiotherapy omission’ phase II study uses PAM-50 as the biomarker to direct treatment [41]. Both the IDEA and PRECISION studies exclude patients above the ages of 69 and 75 years, respectively. The PRECISION group state this exclusion is due to historical difficulties achieving robust follow-up in this population, as well as competing comorbidities interfering with subsequent breast cancer monitoring and evaluation. Finally, EXPERT, ‘a randomised phase III trial of adjuvant radiotherapy versus observation following BCS and endocrine therapy in patients with molecularly characterised luminal A early breast cancer’ is using the biomarker PAM-50 [42]. This is being led by the Australia and New Zealand Breast Cancer Trials Group.

One of the challenges of these studies is that there is no international consensus regarding the level of local recurrence that would be acceptable to clinicians and patients with de-escalation of radiotherapy. The risks and benefits of radiotherapy need to be weighed up for each patient to achieve an individualised treatment decision. Therefore, international consensus on this issue is unlikely. In the PRIMETIME study, a threshold of an ipsilateral breast disease rate ≤4% at 5 years for selective de-escalation of radiotherapy [38] was set, primarily by patient advocates in collaboration with breast cancer clinicians and trialists [38].

## Challenges of Conducting Biomarker-directed De-escalation Studies

De-escalation of therapy studies can be a challenge to set up, conduct and recruit to. Patients may perceive that ‘more is better’ and clinicians may practice to be ‘better safe than sorry’ [19]. It has been found that patients often have quantitative misperceptions regarding adjuvant treatment, overestimate the risk of a negative outcome without treatment and overestimate the positive effect of treatment [43], [44]. Understanding and communicating the risks and benefits of treatments are challenging for both clinicians and patients. Presenting absolute risk rather than relative risk is preferable as the absolute risk describes how likely an event will be (e.g. in one group of patients), whereas the relative risk only describes how much relatively more or less likely an event will be [45]. Education and conveying information regarding patient prognosis and the side-effects of treatment clearly and effectively are essential to enable patients to make informed choices regarding adjuvant treatment options. Greater patient advocate involvement in the development of ‘avoidance of treatment’ studies is important to determine which trial designs are acceptable for patients and also to identify the degree of benefit patients expect before accepting a treatment associated with long-term adverse effects.

There may be considerable financial pressures when considering ‘avoidance of treatment’ studies. There is less incentive for pharmaceutical companies to support de-escalation studies. In countries with privatised medical healthcare systems there may be financial benefits for clinicians to opt for a treatment over ‘avoidance of treatment’ and higher reimbursement may lead to subsequent increased healthcare resource consumption [19]. If there is uncertainty regarding a treatment, physicians may be incentivised to favour treatment over de-escalation [18], [46]. Hospitals are paid per fraction of radiotherapy or cycle of chemotherapy delivered within the UK's National Health Service. Given these financial arrangements, it is important that UK trialists and clinicians engage with commissioners to ensure that ‘de-escalation of treatment’ is not seen to translate into loss of earnings. Commissioners need to be encouraged to support important studies that may ultimately result in much greater health service savings in terms of finance and toxicity.

A Canadian-based study estimated the total savings to a publicly funded healthcare system if omission of radiotherapy became standard in patients with such a low local relapse rate that adjuvant radiotherapy would offer little benefit (i.e. patients ≥60 years with grade I/II T1N0 luminal A; oestrogen receptor/progesterone receptor-positive, HER2-negative and Ki-67 ≤13%). They determined an annual saving of about $2.0 million and $5.1 million if radiotherapy was omitted for all low-risk luminal A breast cancer patients in Ontario and across Canada, respectively. They also estimated that in the UK, savings could be over £14 million [47]. Financial savings must be considered, particularly given the increasing pressures on a government-funded health service without infinite resources.

## Conclusion

The aim is to tailor the need for adjuvant breast radiotherapy, considering each individual patient's risk of local recurrence and the subsequent risk/benefit ratio of radiotherapy. Advances in genomic profiling and IHC may allow delivery of biomarker-directed treatments, which will require assessment within the context of clinical trials. Patient and clinician perceptions regarding the apparent benefit of treatments in specific groups of patients need to be challenged and the concept of ‘avoidance of treatment’ to prevent overtreatment and long-term adverse effects need to be introduced. Misconceptions regarding apparent financial loss with ‘de-escalation of treatment’ studies also need to be addressed. Patient advocate involvement is crucial to these processes. The feasibility of recruitment into biomarker-directed de-escalation studies will become apparent as more studies open. The challenge is to determine if we can accurately risk stratify patients with early breast cancer and avoid the toxicity associated with overtreatment.

## References

[1] Darby S., McGale P., Correa C., Taylor C., Arriagada R., Clarke M. (2011). Effect of radiotherapy after breast-conserving surgery on 10-year recurrence and 15-year breast cancer death: meta-analysis of individual patient data for 10,801 women in 17 randomised trials. Lancet.

[2] Clarke M., Collins R., Darby S., Davies C., Elphinstone P., Evans V. (2005). Effects of radiotherapy and of differences in the extent of surgery for early breast cancer on local recurrence and 15-year survival: an overview of the randomised trials. Lancet.

[3] Braunstein L.Z., Taghian A.G., Niemierko A., Salama L., Capuco A., Bellon J.R. (2017). Breast-cancer subtype, age, and lymph node status as predictors of local recurrence following breast-conserving therapy. Breast Cancer Res Treat.

[4] http://www.sciencedirect.com/science/article/pii/S0140673611616292.

[5] Mannino M., Yarnold J.R. (2009). Local relapse rates are falling after breast conserving surgery and systemic therapy for early breast cancer: can radiotherapy ever be safely withheld?. Radiother Oncol.

[6] Darby S.C., Ewertz M., McGale P., Bennet A.M., Blom-Goldman U., Bronnum D. (2013). Risk of ischemic heart disease in women after radiotherapy for breast cancer. New Engl J Med.

[7] Grantzau T., Overgaard J. (2015). Risk of second non-breast cancer after radiotherapy for breast cancer: a systematic review and meta-analysis of 762,468 patients. Radiother Oncol.

[8] Taylor C., Correa C., Duane F.K., Aznar M.C., Anderson S.J., Bergh J. (2017). Estimating the risks of breast cancer radiotherapy: evidence from modern radiation doses to the lungs and heart and from previous randomized trials. J Clin Oncol.

[9] Haviland J.S., Owen J.R., Dewar J.A., Agrawal R.K., Barrett J., Barrett-Lee P.J. (2013). The UK Standardisation of Breast Radiotherapy (START) trials of radiotherapy hypofractionation for treatment of early breast cancer: 10-year follow-up results of two randomised controlled trials. Lancet Oncol.

[10] Hopwood P., Haviland J.S., Sumo G., Mills J., Bliss J.M., Yarnold J.R. (2010). Comparison of patient-reported breast, arm, and shoulder symptoms and body image after radiotherapy for early breast cancer: 5-year follow-up in the randomised Standardisation of Breast Radiotherapy (START) trials. Lancet Oncol.

[11] Fyles A.W., McCready D.R., Manchul L.A., Trudeau M.E., Merante P., Pintilie M. (2004). Tamoxifen with or without breast irradiation in women 50 years of age or older with early breast cancer. New Engl J Med.

[12] Potter R., Gnant M., Kwasny W., Tausch C., Handl-Zeller L., Pakisch B. (2007). Lumpectomy plus tamoxifen or anastrozole with or without whole breast irradiation in women with favorable early breast cancer. Int J Radiat Oncol Biol Phys.

[13] Hughes K.S., Schnaper L.A., Berry D., Cirrincione C., McCormick B., Shank B. (2004). Lumpectomy plus tamoxifen with or without irradiation in women 70 years of age or older with early breast cancer. New Engl J Med.

[14] Kunkler I.H., Williams L.J., Jack W.J., Cameron D.A., Dixon J.M. (2015). Breast-conserving surgery with or without irradiation in women aged 65 years or older with early breast cancer (PRIME II): a randomised controlled trial. Lancet Oncol.

[15] Blamey R.W., Bates T., Chetty U., Duffy S.W., Ellis I.O., George D. (2013). Radiotherapy or tamoxifen after conserving surgery for breast cancers of excellent prognosis: British Association of Surgical Oncology (BASO) II trial. Eur J Cancer.

[16] Hughes K.S., Schnaper L.A., Bellon J.R., Cirrincione C.T., Berry D.A., McCormick B. (2013). Lumpectomy plus tamoxifen with or without irradiation in women age 70 years or older with early breast cancer: long-term follow-up of CALGB 9343. J Clin Oncol.

[17] Carlson R.W., McCormick B. (2005). Update: NCCN breast cancer clinical practice guidelines. J Natl Comp Cancer Network.

[18] Giordano S.H. (2012). Radiotherapy in older women with low-risk breast cancer: why did practice not change?. J Clin Oncol.

[19] Soulos P.R., Yu J.B., Roberts K.B., Raldow A.C., Herrin J., Long J.B. (2012). Assessing the impact of a cooperative group trial on breast cancer care in the medicare population. J Clin Oncol.

[20] Courdi A., Gerard J.P. (2013). Radiotherapy for elderly patients with breast cancer. J Clin Oncol.

[21] Liu F.F., Shi W., Done S.J., Miller N., Pintilie M., Voduc D. (2015). Identification of a low-risk luminal A breast cancer cohort that may not benefit from breast radiotherapy. J Clin Oncol.

[22] Dowsett M., Cuzick J., Wale C., Forbes J., Mallon E.A., Salter J. (2010). Prediction of risk of distant recurrence using the 21-gene recurrence score in node-negative and node-positive postmenopausal patients with breast cancer treated with anastrozole or tamoxifen: a TransATAC study. J Clin Oncol.

[23] Cuzick J., Dowsett M., Pineda S., Wale C., Salter J., Quinn E. (2011). Prognostic value of a combined estrogen receptor, progesterone receptor, Ki-67, and human epidermal growth factor receptor 2 immunohistochemical score and comparison with the Genomic Health recurrence score in early breast cancer. J Clin Oncol.

[24] Tang G., Cuzick J., Costantino J.P., Dowsett M., Forbes J.F., Crager M. (2011). Risk of recurrence and chemotherapy benefit for patients with node-negative, estrogen receptor-positive breast cancer: recurrence score alone and integrated with pathologic and clinical factors. J Clin Oncol.

[25] Mamounas E.P., Tang G., Fisher B., Paik S., Shak S., Costantino J.P. (2010). Association between the 21-gene recurrence score assay and risk of locoregional recurrence in node-negative, estrogen receptor-positive breast cancer: results from NSABP B-14 and NSABP B-20. J Clin Oncol.

[26] Nielsen T.O., Parker J.S., Leung S., Voduc D., Ebbert M., Vickery T. (2010). A comparison of PAM50 intrinsic subtyping with immunohistochemistry and clinical prognostic factors in tamoxifen-treated estrogen receptor-positive breast cancer. Clin Cancer Res.

[27] Parker J.S., Mullins M., Cheang M.C., Leung S., Voduc D., Vickery T. (2009). Supervised risk predictor of breast cancer based on intrinsic subtypes. J Clin Oncol.

[28] Gnant M., Filipits M., Greil R., Stoeger H., Rudas M., Bago-Horvath Z., Mlineritsch B. (2014). Predicting distant recurrence in receptor-positive breast cancer patients with limited clinicopathological risk: using the PAM50 Risk of Recurrence score in 1478 postmenopausal patients of the ABCSG-8 trial treated with adjuvant endocrine therapy alone. Ann Oncol.

[29] Dowsett M., Sestak I., Lopez-Knowles E., Sidhu K., Dunbier A.K., Cowens J.W. (2013). Comparison of PAM50 risk of recurrence score with oncotype DX and IHC4 for predicting risk of distant recurrence after endocrine therapy. J Clin Oncol.

[30] Sestak I., Cuzick J., Dowsett M., Lopez-Knowles E., Filipits M., Dubsky P. (2015). Prediction of late distant recurrence after 5 years of endocrine treatment: a combined analysis of patients from the Austrian breast and colorectal cancer study group 8 and arimidex, tamoxifen alone or in combination randomized trials using the PAM50 risk of recurrence score. J Clin Oncol.

[31] Fitzal F., Fesl C., Rudas M., Dubsky P.C., Bartsch R. (2014). Predicting local recurrence using PAM50 in postmenopausal endocrine responsive breast cancer patients. J Clin Oncol.

[32] Fitzal F., Filipits M., Rudas M., Greil R., Dietze O., Samonigg H. (2015). The genomic expression test EndoPredict is a prognostic tool for identifying risk of local recurrence in postmenopausal endocrine receptor-positive, her2neu-negative breast cancer patients randomised within the prospective ABCSG 8 trial. Br J Cancer.

[33] Cuzick J., Sestak I., Baum M., Buzdar A., Howell A., Dowsett M. (2010). Effect of anastrozole and tamoxifen as adjuvant treatment for early-stage breast cancer: 10-year analysis of the ATAC trial. Lancet Oncol.

[34] Lakhanpal R., Sestak I., Shadbolt B., Bennett G.M., Brown M., Phillips T. (2016). IHC4 score plus clinical treatment score predicts locoregional recurrence in early breast cancer. Breast.

[37] Prescott R.J., Kunkler I.H., Williams L.J., King C.C., Jack W., van der Pol M. (2007). A randomised controlled trial of postoperative radiotherapy following breast-conserving surgery in a minimum-risk older population. The PRIME trial. Health Technol Assess.

[38] Kirwan C.C., Coles C.E., Bliss J. (2016). It's PRIMETIME. Postoperative avoidance of radiotherapy: biomarker selection of women at very low risk of local recurrence. Clin Oncol.

[39] LUMINA. Available at: https://clinicaltrials.gov/ct2/show/NCT01791829?term=LUMINA+breast+cancer&rank=1.

[40] IDEA. Available at: https://clinicaltrials.gov/ct2/show/NCT02400190?term=IDEA+breast+cancer&rank=1.

[41] PRECISION. Available at: https://clinicaltrials.gov/ct2/show/NCT02653755?term=PRECISION+breast+cancer&rank=1.

[42] EXPERT trials. Available at: https://clinicaltrials.gov/ct2/show/NCT02889874.

[43] Ravdin P.M., Siminoff I.A., Harvey J.A. (1998). Survey of breast cancer patients concerning their knowledge and expectations of adjuvant therapy. J Clin Oncol.

[44] Fetting J.H., Siminoff L.A., Piantadosi S., Abeloff M.D., Damron D.J., Sarsfield A.M. (1990). Effect of patients' expectations of benefit with standard breast cancer adjuvant chemotherapy on participation in a randomized clinical trial: a clinical vignette study. J Clin Oncol.

[45] Baglin T. (2009). Communicating benefit and risk. Br J Haematol.

[46] Shen J., Andersen R., Brook R., Kominski G., Albert P.S., Wenger N. (2004). The effects of payment method on clinical decision-making: physician responses to clinical scenarios. Med Care.

[47] Han K., Yap M.L., Yong J.H., Mittmann N., Hoch J.S., Fyles A.W. (2016). Omission of breast radiotherapy in low-risk luminal A breast cancer: impact on health care costs. Clin Oncol.

